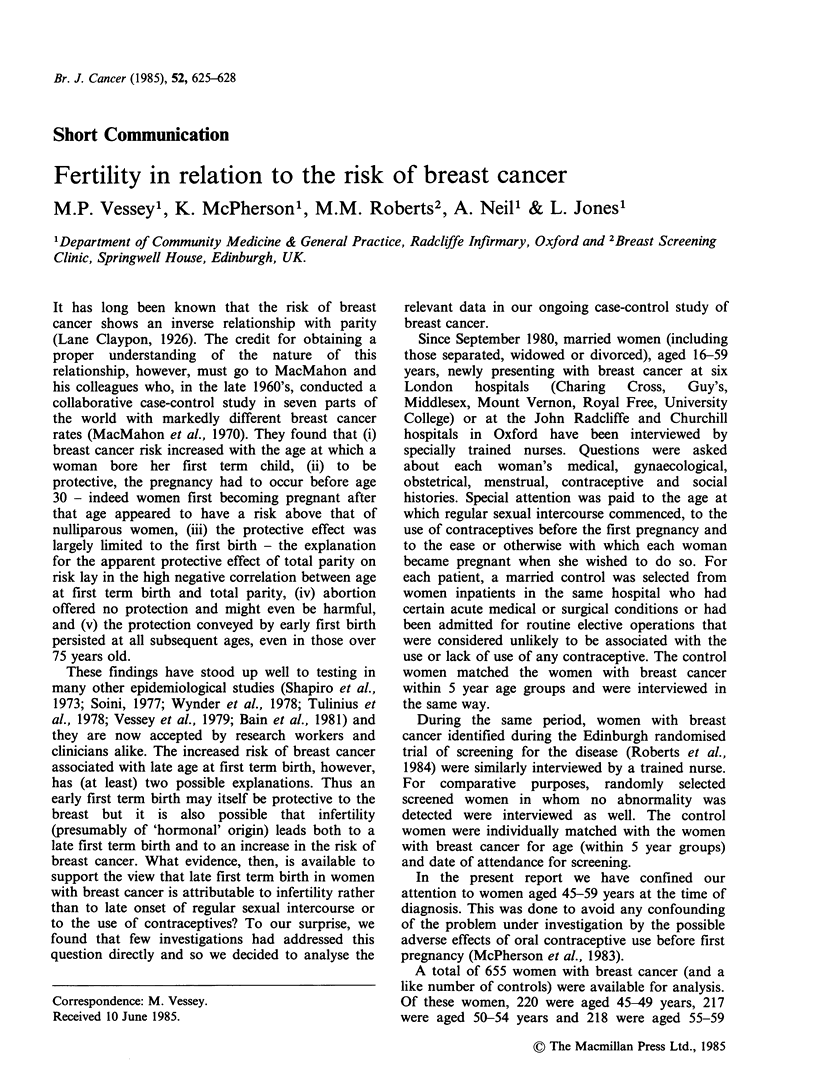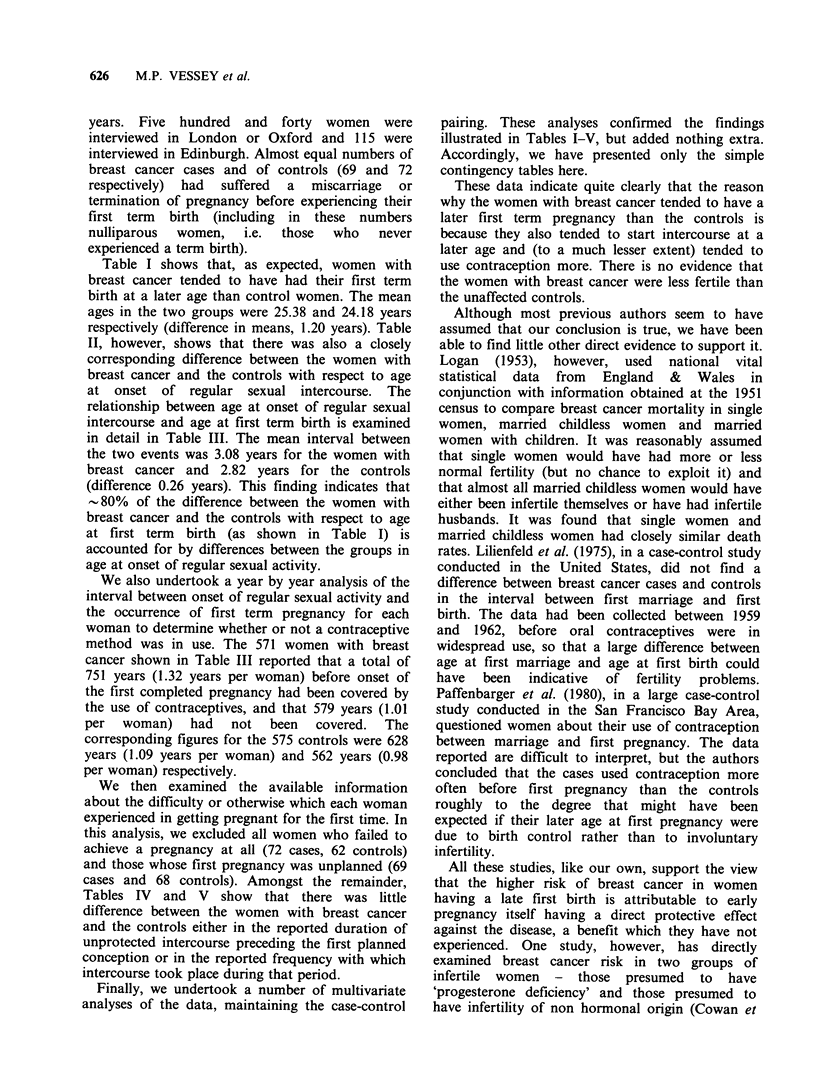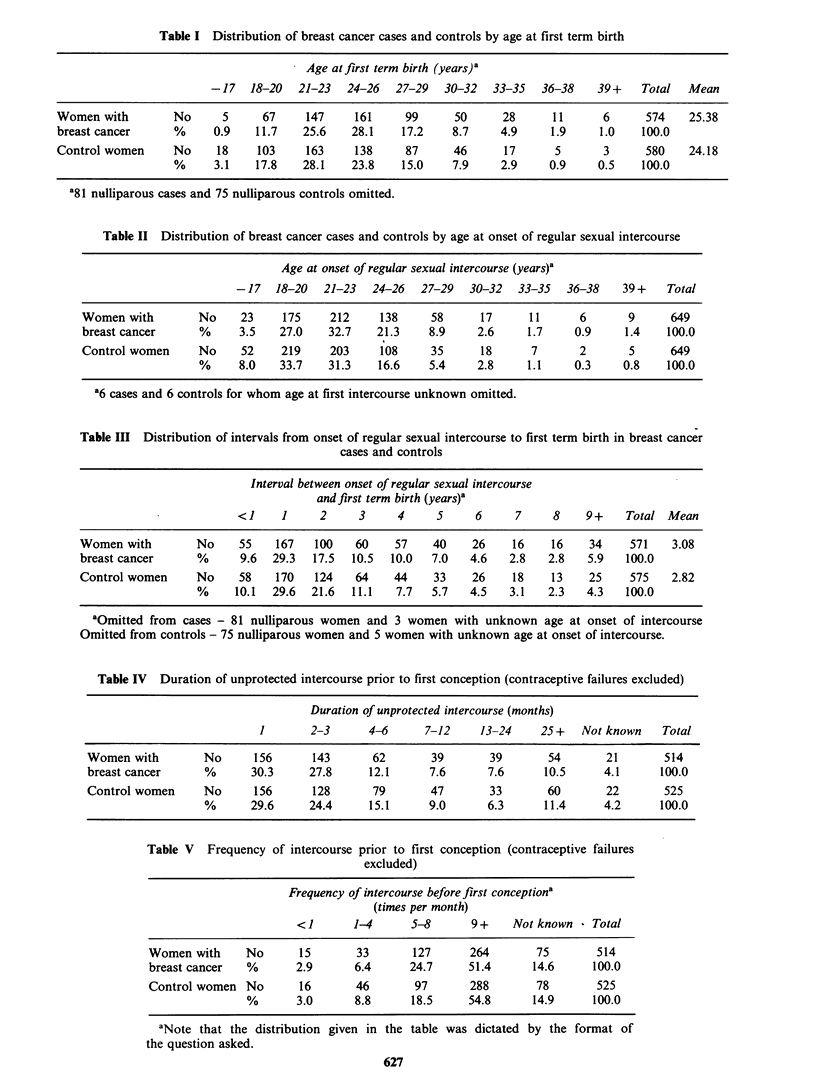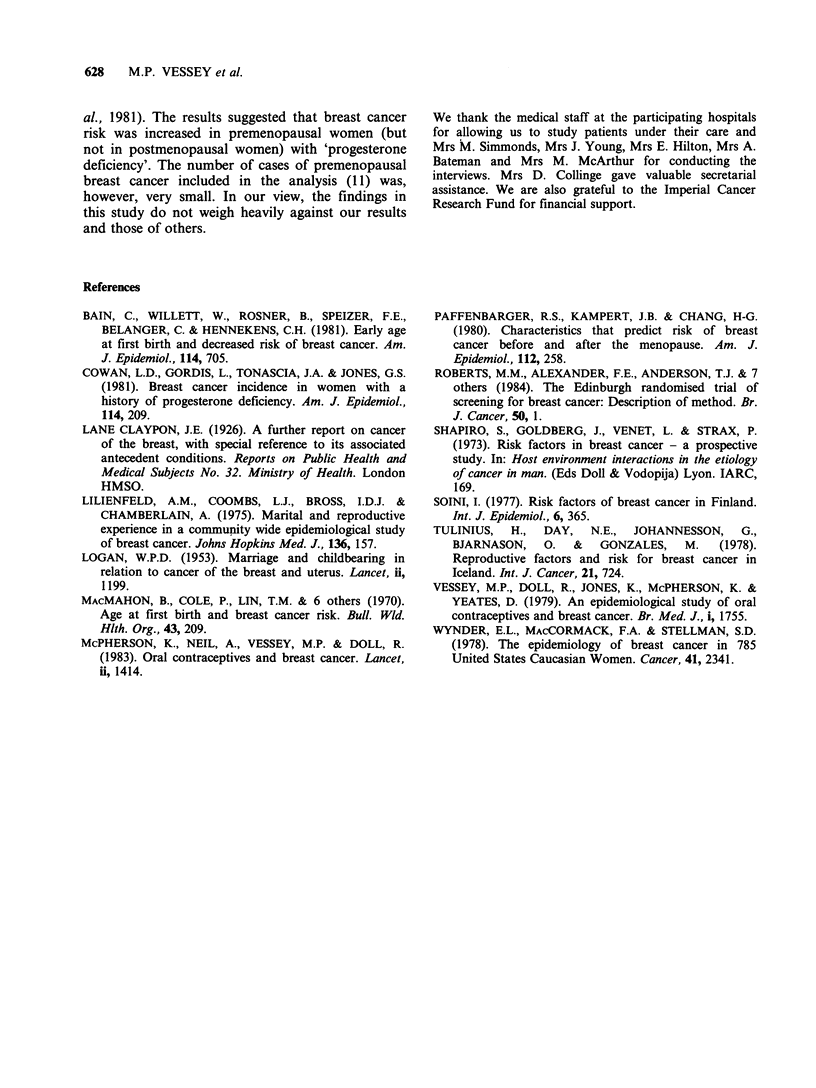# Fertility in relation to the risk of breast cancer.

**DOI:** 10.1038/bjc.1985.236

**Published:** 1985-10

**Authors:** M. P. Vessey, K. McPherson, M. M. Roberts, A. Neil, L. Jones


*Br. J. Cancer* (1985), **52,** 625–628

### Short Communication

### Fertility in relation to the risk of breast cancer

M.P. Vessey[1], K. McPherson[1], M.M. Roberts[2], A. Neil[1] & L. Jones[1]

[1]*Department of Community Medicine & General Practice, Radcliffe Infirmary, Oxford and* [2]*Breast Screening Clinic, Springwell House, Edinburgh, UK.*

It has long been known that the risk of breast cancer shows an inverse relationship with parity (Lane Claypon, 1926). The credit for obtaining a proper understanding of the nature of this relationship, however, must go to MacMahon and his colleagues who, in the late 1960's, conducted a collaborative case-control study in seven parts of the world with markedly different breast cancer rates (MacMahon *et al.*, 1970). They found that (i) breast cancer risk increased with the age at which a woman bore her first term child, (ii) to be protective, the pregnancy had to occur before age 30 – indeed women first becoming pregnant after that age appeared to have a risk above that of nulliparous women, (iii) the protective effect was largely limited to the first birth – the explanation for the apparent protective effect of total parity on risk lay in the high negative correlation between age at first term birth and total parity, (iv) abortion offered no protection and might even be harmful, and (v) the protection conveyed by early first birth persisted at all subsequent ages, even in those over 75 years old.

These findings have stood up well to testing in many other epidemiological studies (Shapiro *et al.*, 1973; Soini, 1977; Wynder *et al.*, 1978; Tulinius *et al.*, 1978; Vessey *et al.*, 1979; Bain *et al.*, 1981) and they are now accepted by research workers and clinicians alike. The increased risk of breast cancer associated with late age at first term birth, however, has (at least) two possible explanations. Thus an early first term birth may itself be protective to the breast but it is also possible that infertility (presumably of 'hormonal' origin) leads both to a late first term birth and to an increase in the risk of breast cancer. What evidence, then, is available to support the view that late first term birth in women with breast cancer is attributable to infertility rather than to late onset of regular sexual intercourse or to the use of contraceptives? To our surprise, we found that few investigations had addressed this question directly and so we decided to analyse the relevant data in our ongoing case-control study of breast cancer.

Correspondence: M. Vessey.
Received 10 June 1985.

Since September 1980, married women (including those separated, widowed or divorced), aged 16–59 years, newly presenting with breast cancer at six London hospitals (Charing Cross, Guy's, Middlesex, Mount Vernon, Royal Free, University College) or at the John Radcliffe and Churchill hospitals in Oxford have been interviewed by specially trained nurses. Questions were asked about each woman's medical, gynaecological, obstetrical, menstrual, contraceptive and social histories. Special attention was paid to the age at which regular sexual intercourse commenced, to the use of contraceptives before the first pregnancy and to the ease or otherwise with which each woman became pregnant when she wished to do so. For each patient, a married control was selected from women inpatients in the same hospital who had certain acute medical or surgical conditions or had been admitted for routine elective operations that were considered unlikely to be associated with the use or lack of use of any contraceptive. The control women matched the women with breast cancer within 5 year age groups and were interviewed in the same way.

During the same period, women with breast cancer identified during the Edinburgh randomised trial of screening for the disease (Roberts *et al.*, 1984) were similarly interviewed by a trained nurse. For comparative purposes, randomly selected screened women in whom no abnormality was detected were interviewed as well. The control women were individually matched with the women with breast cancer for age (within 5 year groups) and date of attendance for screening.

In the present report we have confined our attention to women aged 45–59 years at the time of diagnosis. This was done to avoid any confounding of the problem under investigation by the possible adverse effects of oral contraceptive use before first pregnancy (McPherson *et al.*, 1983).

A total of 655 women with breast cancer (and a like number of controls) were available for analysis. Of these women, 220 were aged 45–49 years, 217 were aged 50–54 years and 218 were aged 55–59

© The Macmillan Press Ltd., 1985

years. Five hundred and forty women were interviewed in London or Oxford and 115 were interviewed in Edinburgh. Almost equal numbers of breast cancer cases and of controls (69 and 72 respectively) had suffered a miscarriage or termination of pregnancy before experiencing their first term birth (including in these numbers nulliparous women, i.e. those who never experienced a term birth).

Table I shows that, as expected, women with breast cancer tended to have had their first term birth at a later age than control women. The mean ages in the two groups were 25.38 and 24.18 years respectively (difference in means, 1.20 years). Table II, however, shows that there was also a closely corresponding difference between the women with breast cancer and the controls with respect to age at onset of regular sexual intercourse. The relationship between age at onset of regular sexual intercourse and age at first term birth is examined in detail in Table III. The mean interval between the two events was 3.08 years for the women with breast cancer and 2.82 years for the controls (difference 0.26 years). This finding indicates that ~80% of the difference between the women with breast cancer and the controls with respect to age at first term birth (as shown in Table I) is accounted for by differences between the groups in age at onset of regular sexual activity.

We also undertook a year by year analysis of the interval between onset of regular sexual activity and the occurrence of first term pregnancy for each woman to determine whether or not a contraceptive method was in use. The 571 women with breast cancer shown in Table III reported that a total of 751 years (1.32 years per woman) before onset of the first completed pregnancy had been covered by the use of contraceptives, and that 579 years (1.01 per woman) had not been covered. The corresponding figures for the 575 controls were 628 years (1.09 years per woman) and 562 years (0.98 per woman) respectively.

We then examined the available information about the difficulty or otherwise which each woman experienced in getting pregnant for the first time. In this analysis, we excluded all women who failed to achieve a pregnancy at all (72 cases, 62 controls) and those whose first pregnancy was unplanned (69 cases and 68 controls). Amongst the remainder, Tables IV and V show that there was little difference between the women with breast cancer and the controls either in the reported duration of unprotected intercourse preceding the first planned conception or in the reported frequency with which intercourse took place during that period.

Finally, we undertook a number of multivariate analyses of the data, maintaining the case-control pairing. These analyses confirmed the findings illustrated in Tables I–V, but added nothing extra. Accordingly, we have presented only the simple contingency tables here.

These data indicate quite clearly that the reason why the women with breast cancer tended to have a later first term pregnancy than the controls is because they also tended to start intercourse at a later age and (to a much lesser extent) tended to use contraception more. There is no evidence that the women with breast cancer were less fertile than the unaffected controls.

Although most previous authors seem to have assumed that our conclusion is true, we have been able to find little other direct evidence to support it. Logan (1953), however, used national vital statistical data from England & Wales in conjunction with information obtained at the 1951 census to compare breast cancer mortality in single women, married childless women and married women with children. It was reasonably assumed that single women would have had more or less normal fertility (but no chance to exploit it) and that almost all married childless women would have either been infertile themselves or have had infertile husbands. It was found that single women and married childless women had closely similar death rates. Lilienfeld *et al.* (1975), in a case-control study conducted in the United States, did not find a difference between breast cancer cases and controls in the interval between first marriage and first birth. The data had been collected between 1959 and 1962, before oral contraceptives were in widespread use, so that a large difference between age at first marriage and age at first birth could have been indicative of fertility problems. Paffenbarger *et al.* (1980), in a large case-control study conducted in the San Francisco Bay Area, questioned women about their use of contraception between marriage and first pregnancy. The data reported are difficult to interpret, but the authors concluded that the cases used contraception more often before first pregnancy than the controls roughly to the degree that might have been expected if their later age at first pregnancy were due to birth control rather than to involuntary infertility.

All these studies, like our own, support the view that the higher risk of breast cancer in women having a late first birth is attributable to early pregnancy itself having a direct protective effect against the disease, a benefit which they have not experienced. One study, however, has directly examined breast cancer risk in two groups of infertile women – those presumed to have 'progesterone deficiency' and those presumed to have infertility of non hormonal origin (Cowan *et*

**Table I** Distribution of breast cancer cases and controls by age at first term birth

| | | *Age at first term birth (years)*[a] | | | | | | | | | | |
|---|---|---|---|---|---|---|---|---|---|---|---|---|
| | | *−17* | *18–20* | *21–23* | *24–26* | *27–29* | *30–32* | *33–35* | *36–38* | *39+* | *Total* | *Mean* |
| Women with breast cancer | No | 5 | 67 | 147 | 161 | 99 | 50 | 28 | 11 | 6 | 574 | 25.38 |
| | % | 0.9 | 11.7 | 25.6 | 28.1 | 17.2 | 8.7 | 4.9 | 1.9 | 1.0 | 100.0 | |
| Control women | No | 18 | 103 | 163 | 138 | 87 | 46 | 17 | 5 | 3 | 580 | 24.18 |
| | % | 3.1 | 17.8 | 28.1 | 23.8 | 15.0 | 7.9 | 2.9 | 0.9 | 0.5 | 100.0 | |

[a]81 nulliparous cases and 75 nulliparous controls omitted.

**Table II** Distribution of breast cancer cases and controls by age at onset of regular sexual intercourse

| | | *Age at onset of regular sexual intercourse (years)*[a] | | | | | | | | | |
|---|---|---|---|---|---|---|---|---|---|---|---|
| | | *−17* | *18–20* | *21–23* | *24–26* | *27–29* | *30–32* | *33–35* | *36–38* | 39+ | *Total* |
| Women with breast cancer | No | 23 | 175 | 212 | 138 | 58 | 17 | 11 | 6 | 9 | 649 |
| | % | 3.5 | 27.0 | 32.7 | 21.3 | 8.9 | 2.6 | 1.7 | 0.9 | 1.4 | 100.0 |
| Control women | No | 52 | 219 | 203 | 108 | 35 | 18 | 7 | 2 | 5 | 649 |
| | % | 8.0 | 33.7 | 31.3 | 16.6 | 5.4 | 2.8 | 1.1 | 0.3 | 0.8 | 100.0 |

[a]6 cases and 6 controls for whom age at first intercourse unknown omitted.

**Table III** Distribution of intervals from onset of regular sexual intercourse to first term birth in breast cancer cases and controls

| | | *Interval between onset of regular sexual intercourse and first term birth (years)*[a] | | | | | | | | | | |
|---|---|---|---|---|---|---|---|---|---|---|---|---|
| | | *<1* | *1* | *2* | *3* | *4* | *5* | *6* | *7* | *8* | *9+* | *Total* | *Mean* |
| Women with breast cancer | No | 55 | 167 | 100 | 60 | 57 | 40 | 26 | 16 | 16 | 34 | 571 | 3.08 |
| | % | 9.6 | 29.3 | 17.5 | 10.5 | 10.0 | 7.0 | 4.6 | 2.8 | 2.8 | 5.9 | 100.0 | |
| Control women | No | 58 | 170 | 124 | 64 | 44 | 33 | 26 | 18 | 13 | 25 | 575 | 2.82 |
| | % | 10.1 | 29.6 | 21.6 | 11.1 | 7.7 | 5.7 | 4.5 | 3.1 | 2.3 | 4.3 | 100.0 | |

[a]Omitted from cases – 81 nulliparous women and 3 women with unknown age at onset of intercourse Omitted from controls – 75 nulliparous women and 5 women with unknown age at onset of intercourse.

**Table IV** Duration of unprotected intercourse prior to first conception (contraceptive failures excluded)

| | | *Duration of unprotected intercourse (months)* | | | | | | | |
|---|---|---|---|---|---|---|---|---|---|
| | | *1* | *2–3* | *4–6* | *7–12* | *13–24* | *25+* | *Not known* | *Total* |
| Women with breast cancer | No | 156 | 143 | 62 | 39 | 39 | 54 | 21 | 514 |
| | % | 30.3 | 27.8 | 12.1 | 7.6 | 7.6 | 10.5 | 4.1 | 100.0 |
| Control women | No | 156 | 128 | 79 | 47 | 33 | 60 | 22 | 525 |
| | % | 29.6 | 24.4 | 15.1 | 9.0 | 6.3 | 11.4 | 4.2 | 100.0 |

**Table V** Frequency of intercourse prior to first conception (contraceptive failures excluded)

| | | *Frequency of intercourse before first conception*[a] *(times per month)* | | | | | |
|---|---|---|---|---|---|---|---|
| | | *<1* | *1–4* | *5–8* | *9+* | *Not known* | *Total* |
| Women with breast cancer | No | 15 | 33 | 127 | 264 | 75 | 514 |
| | % | 2.9 | 6.4 | 24.7 | 51.4 | 14.6 | 100.0 |
| Control women | No | 16 | 46 | 97 | 288 | 78 | 525 |
| | % | 3.0 | 8.8 | 18.5 | 54.8 | 14.9 | 100.0 |

[a]Note that the distribution given in the table was dictated by the format of the question asked.

al., 1981). The results suggested that breast cancer risk was increased in premenopausal women (but not in postmenopausal women) with 'progesterone deficiency'. The number of cases of premenopausal breast cancer included in the analysis (11) was, however, very small. In our view, the findings in this study do not weigh heavily against our results and those of others.

We thank the medical staff at the participating hospitals for allowing us to study patients under their care and Mrs M. Simmonds, Mrs J. Young, Mrs E. Hilton, Mrs A. Bateman and Mrs M. McArthur for conducting the interviews. Mrs D. Collinge gave valuable secretarial assistance. We are also grateful to the Imperial Cancer Research Fund for financial support.

### References

BAIN, C., WILLETT, W., ROSNER, B., SPEIZER, F.E., BELANGER, C. & HENNEKENS, C.H. (1981). Early age at first birth and decreased risk of breast cancer. *Am. J. Epidemiol.*, **114**, 705.

COWAN, L.D., GORDIS, L., TONASCIA, J.A. & JONES, G.S. (1981). Breast cancer incidence in women with a history of progesterone deficiency. *Am. J. Epidemiol.*, **114**, 209.

LANE CLAYPON, J.E. (1926). A further report on cancer of the breast, with special reference to its associated antecedent conditions. *Reports on Public Health and Medical Subjects No. 32. Ministry of Health.* London HMSO.

LILIENFELD, A.M., COOMBS, L.J., BROSS, I.D.J. & CHAMBERLAIN, A. (1975). Marital and reproductive experience in a community wide epidemiological study of breast cancer. *Johns Hopkins Med. J.*, **136**, 157.

LOGAN, W.P.D. (1953). Marriage and childbearing in relation to cancer of the breast and uterus. *Lancet*, **ii**, 1199.

MACMAHON, B., COLE, P., LIN, T.M. & 6 others (1970). Age at first birth and breast cancer risk. *Bull. Wld. Hlth. Org.*, **43**, 209.

MCPHERSON, K., NEIL, A., VESSEY, M.P. & DOLL, R. (1983). Oral contraceptives and breast cancer. *Lancet*, **ii**, 1414.

PAFFENBARGER, R.S., KAMPERT, J.B. & CHANG, H-G. (1980). Characteristics that predict risk of breast cancer before and after the menopause. *Am. J. Epidemiol.*, **112**, 258.

ROBERTS, M.M., ALEXANDER, F.E., ANDERSON, T.J. & 7 others (1984). The Edinburgh randomised trial of screening for breast cancer: Description of method. *Br. J. Cancer*, **50**, 1.

SHAPIRO, S., GOLDBERG, J., VENET, L. & STRAX, P. (1973). Risk factors in breast cancer – a prospective study. In: *Host environment interactions in the etiology of cancer in man.* (Eds Doll & Vodopija) Lyon. IARC, 169.

SOINI, I. (1977). Risk factors of breast cancer in Finland. *Int. J. Epidemiol.*, **6**, 365.

TULINIUS, H., DAY, N.E., JOHANNESSON, G., BJARNASON, O. & GONZALES, M. (1978). Reproductive factors and risk for breast cancer in Iceland. *Int. J. Cancer*, **21**, 724.

VESSEY, M.P., DOLL, R., JONES, K., MCPHERSON, K. & YEATES, D. (1979). An epidemiological study of oral contraceptives and breast cancer. *Br. Med. J.*, **i**, 1755.

WYNDER, E.L., MACCORMACK, F.A. & STELLMAN, S.D. (1978). The epidemiology of breast cancer in 785 United States Caucasian Women. *Cancer*, **41**, 2341.